# (*Z*)-2-Amino-3-[(*E*)-benzyl­ideneamino]but-2-enedinitrile

**DOI:** 10.1107/S1600536809010873

**Published:** 2009-03-28

**Authors:** G. Varsha, V. Arun, Manju Sebastian, P. Leeju, Digna Varghese, K. K. M. Yusuff

**Affiliations:** a Department of Applied Chemistry, Cochin University of Science and Technology, Kochi 682 022, Kerala, India

## Abstract

The asymmetric unit of the title compound, C_11_H_8_N_4_, contains two independent mol­ecules. In the crystal structure, inter­molecular N—H⋯N hydrogen bonds link mol­ecules into ribbons extended in the [100] direction.

## Related literature

For some properties of Schiff base ligands, see: Arun, Robinson *et al.* (2009 ▶); Arun, Sridevi *et al.* (2009 ▶). For related structures, see: MacLachlan *et al.* (1996 ▶); Mague & Eduok (2000 ▶); Varghese *et al.* (2009 ▶).
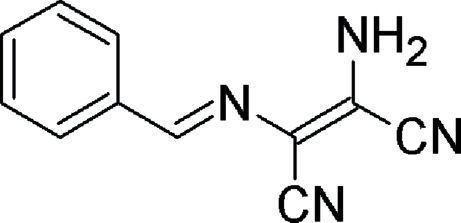


## Experimental

### 

#### Crystal data

C_11_H_8_N_4_
*M* *_r_* = 196.21
Monoclinic, 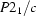

*a* = 6.9569 (19) Å
*b* = 22.796 (6) Å
*c* = 13.516 (4) Å
β = 100.983 (5)°
*V* = 2104.2 (10) Å^3^
*Z* = 8
Mo *K*α radiation radiation
μ = 0.08 mm^−1^
*T* = 298 K
0.42 × 0.18 × 0.18 mm

#### Data collection

Bruker SMART APEX CCD area-detector diffractometer
Absorption correction: multi-scan (*SADABS*; Sheldrick, 2001 ▶) *T*_min_ = 0.980, *T*_max_ = 0.984
12239 measured reflections
4173 independent reflections
3216 reflections with *I* > 2σ(*I*)
*R*_int_ = 0.036

#### Refinement

*R*[*F* ^2^ > 2σ(*F* ^2^)] = 0.106
*wR*(*F* ^2^) = 0.214
*S* = 1.29
4173 reflections
272 parameters
H-atom parameters constrained
Δρ_max_ = 0.22 e Å^−3^
Δρ_min_ = −0.21 e Å^−3^

### 

Data collection: *SMART* (Bruker, 2000 ▶); cell refinement: *SAINT* (Bruker, 2000 ▶); data reduction: *SAINT*; program(s) used to solve structure: *SHELXTL* (Sheldrick, 2008 ▶); program(s) used to refine structure: *SHELXTL*; molecular graphics: *SHELXTL*; software used to prepare material for publication: *SHELXTL* and *PLATON* (Spek, 2009 ▶).

## Supplementary Material

Crystal structure: contains datablocks I, global. DOI: 10.1107/S1600536809010873/cv2533sup1.cif

Structure factors: contains datablocks I. DOI: 10.1107/S1600536809010873/cv2533Isup2.hkl

Additional supplementary materials:  crystallographic information; 3D view; checkCIF report

## Figures and Tables

**Table 1:** Hydrogen-bond geometry (Å, °)

*D*—H⋯*A*	*D*—H	H⋯*A*	*D*⋯*A*	*D*—H⋯*A*
N2—H2*A*⋯N3^i^	0.86	2.36	3.096 (4)	144
N2—H2*B*⋯N8^ii^	0.86	2.25	3.090 (5)	165
N6—H6*A*⋯N7^iii^	0.86	2.51	3.228 (5)	142
N6—H6*B*⋯N4^iv^	0.86	2.28	3.057 (4)	150

Symmetry codes: (i) 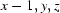; (ii) 
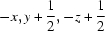
; (iii) 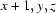; (iv) 
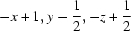
.

**Fig. 1.**
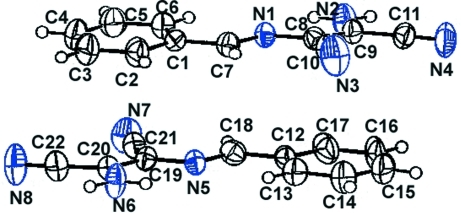
Two independent molecules of (I) with the atomic labelling scheme and 50% probability displacement ellipsoids.

## supplementary crystallographic information

### Comment

In continuation of our study of Schiff base ligands and their metal complexes (Arun, Robinson *et al.*, 2009; Arun, Sridevi *et al.*, 2009; Varghese *et al.*, 2009), we present here the title compound, (I).

The asymmetric unit of (I) contains two independent molecules (Fig. 1). All bond lengths and angles in (I) are normal and correspond to those observed in the related compounds (MacLachlan *et al.*, 1996; Mague & Eduok 2000). In both independent molecules, the benzene ring and diaminomaleonitrile moiety are anti with respect to azomethine C=N. In the crystal structure, intermolecular N—H···N hydrogen bonds (Table 1) link the molecules into ribbons extended in direction [100].

### Experimental

Benzaldehyde (Merck) and 2,3-diaminomaleonitrile (Aldrich) are of reagent grade and are used without further purification. A hot solution of 2,3-diaminomaleonitrile (1 mmol) in methanol (25 ml) was added slowly over a hot solution of benzaldehyde (1 mmol) in the same solvent (25 ml) and the solution was refluxed for three hours. The resulting yellow solution was cooled in ice and the precipitated imine was filtered off and washed with cold methanol and dried under vacuum. Yellow crystals of (1) suitable for X-ray analysis were obtained by slow evaporation of the Schiff base in absolute ethanol (yield 85%; m.p. 463 K).

### Refinement

H atoms were positioned geometrically (C—H = 0.93 Å, N—H = 0.86 Å) and refined in riding mode, with *U*_iso_ (H) = 1.2Ueq(C, N).

### Crystal data

**Table:**

C_11_H_8_N_4_	*F*(000) = 816
*M**_r_* = 196.21	*D*_x_ = 1.239 Mg m^−^^3^
Monoclinic, *P*2_1_/*c*	Mo *K*α radiation radiation, λ = 0.71073 Å
Hall symbol: -P 2ybc	Cell parameters from 4803 reflections
*a* = 6.9569 (19) Å	θ = 2.4–26.1°
*b* = 22.796 (6) Å	µ = 0.08 mm^−^^1^
*c* = 13.516 (4) Å	*T* = 298 K
β = 100.983 (5)°	Rod, yellow
*V* = 2104.2 (10) Å^3^	0.42 × 0.18 × 0.18 mm
*Z* = 8	

### Data collection

**Table:**

Bruker SMART APEX CCD area-detector diffractometer	4173 independent reflections
Radiation source: fine-focus sealed tube	3216 reflections with *I* > 2σ(*I*)
graphite	*R*_int_ = 0.036
φ and ω scans	θ_max_ = 26.1°, θ_min_ = 1.8°
Absorption correction: multi-scan (*SADABS*; Sheldrick, 2001)	*h* = −8→8
*T*_min_ = 0.980, *T*_max_ = 0.984	*k* = −28→23
12239 measured reflections	*l* = −16→15

### Refinement

**Table:**

Refinement on *F*^2^	Secondary atom site location: difference Fourier map
Least-squares matrix: full	Hydrogen site location: inferred from neighbouring sites
*R*[*F*^2^ > 2σ(*F*^2^)] = 0.106	H-atom parameters constrained
*wR*(*F*^2^) = 0.214	*w* = 1/[σ^2^(*F*_o_^2^) + (0.0476*P*)^2^ + 1.6604*P*] where *P* = (*F*_o_^2^ + 2*F*_c_^2^)/3
*S* = 1.29	(Δ/σ)_max_ < 0.001
4173 reflections	Δρ_max_ = 0.22 e Å^−^^3^
272 parameters	Δρ_min_ = −0.21 e Å^−^^3^
0 restraints	Extinction correction: *SHELXTL* (Sheldrick, 2008), Fc^*^=kFc[1+0.001xFc^2^λ^3^/sin(2θ)]^-1/4^
Primary atom site location: structure-invariant direct methods	Extinction coefficient: 0.0021 (6)

### Special details

**Table:**

Geometry. All e.s.d.'s (except the e.s.d. in the dihedral angle between two l.s. planes) are estimated using the full covariance matrix. The cell e.s.d.'s are taken into account individually in the estimation of e.s.d.'s in distances, angles and torsion angles; correlations between e.s.d.'s in cell parameters are only used when they are defined by crystal symmetry. An approximate (isotropic) treatment of cell e.s.d.'s is used for estimating e.s.d.'s involving l.s. planes.
Refinement. Refinement of *F*^2^ against ALL reflections. The weighted *R*-factor *wR* and goodness of fit *S* are based on *F*^2^, conventional *R*-factors *R* are based on *F*, with *F* set to zero for negative *F*^2^. The threshold expression of *F*^2^ > σ(*F*^2^) is used only for calculating *R*-factors(gt) *etc*. and is not relevant to the choice of reflections for refinement. *R*-factors based on *F*^2^ are statistically about twice as large as those based on *F*, and *R*- factors based on ALL data will be even larger.

### Fractional atomic coordinates and isotropic or equivalent isotropic displacement parameters (Å^2^)

**Table:**

	*x*	*y*	*z*	*U*_iso_*/*U*_eq_	
C1	0.3264 (5)	0.39467 (14)	0.3626 (2)	0.0398 (8)	
C2	0.3971 (6)	0.34110 (16)	0.3379 (3)	0.0590 (11)	
H2	0.5196	0.3387	0.3202	0.071*	
C3	0.2851 (8)	0.29092 (19)	0.3397 (4)	0.0772 (14)	
H3	0.3324	0.2548	0.3229	0.093*	
C4	0.1054 (8)	0.2944 (2)	0.3661 (4)	0.0792 (15)	
H4	0.0313	0.2606	0.3677	0.095*	
C5	0.0339 (6)	0.3473 (2)	0.3902 (3)	0.0673 (12)	
H5	−0.0887	0.3494	0.4078	0.081*	
C6	0.1427 (5)	0.39773 (17)	0.3883 (3)	0.0501 (9)	
H6	0.0933	0.4337	0.4043	0.060*	
C7	0.4454 (5)	0.44731 (14)	0.3591 (3)	0.0388 (8)	
H7	0.5684	0.4436	0.3422	0.047*	
C8	0.5013 (4)	0.54703 (14)	0.3760 (2)	0.0366 (8)	
C9	0.4227 (5)	0.60037 (14)	0.3899 (3)	0.0397 (8)	
C10	0.6996 (5)	0.54341 (15)	0.3605 (3)	0.0469 (9)	
C11	0.5356 (5)	0.65316 (15)	0.3859 (3)	0.0459 (9)	
N1	0.3855 (4)	0.49769 (11)	0.3784 (2)	0.0367 (7)	
N2	0.2412 (4)	0.60811 (13)	0.4084 (2)	0.0558 (9)	
H2A	0.1673	0.5783	0.4122	0.067*	
H2B	0.1991	0.6429	0.4165	0.067*	
N3	0.8559 (5)	0.53781 (16)	0.3485 (3)	0.0764 (12)	
N4	0.6208 (5)	0.69534 (14)	0.3808 (3)	0.0693 (11)	
C12	0.2008 (5)	0.53605 (15)	0.1295 (3)	0.0452 (9)	
C13	0.3849 (5)	0.53053 (17)	0.1052 (3)	0.0550 (10)	
H13	0.4312	0.4934	0.0933	0.066*	
C14	0.4994 (7)	0.5783 (2)	0.0985 (4)	0.0736 (13)	
H14	0.6224	0.5739	0.0819	0.088*	
C15	0.4310 (8)	0.6331 (2)	0.1165 (4)	0.0823 (16)	
H15	0.5078	0.6659	0.1109	0.099*	
C16	0.2515 (9)	0.64021 (18)	0.1425 (4)	0.0821 (16)	
H16	0.2090	0.6775	0.1562	0.098*	
C17	0.1334 (7)	0.59197 (17)	0.1484 (3)	0.0639 (12)	
H17	0.0104	0.5967	0.1648	0.077*	
C18	0.0775 (5)	0.48496 (15)	0.1349 (3)	0.0450 (9)	
H18	−0.0432	0.4899	0.1538	0.054*	
C19	0.0133 (4)	0.38528 (14)	0.1179 (3)	0.0379 (8)	
C20	0.0863 (5)	0.33203 (15)	0.0987 (3)	0.0416 (8)	
C21	−0.1801 (5)	0.38947 (15)	0.1396 (3)	0.0450 (9)	
C22	−0.0336 (6)	0.28020 (17)	0.0983 (3)	0.0540 (10)	
N5	0.1316 (4)	0.43361 (12)	0.1143 (2)	0.0396 (7)	
N6	0.2648 (4)	0.32364 (12)	0.0767 (2)	0.0555 (9)	
H6A	0.3410	0.3531	0.0741	0.067*	
H6B	0.3028	0.2888	0.0652	0.067*	
N7	−0.3334 (4)	0.39419 (15)	0.1568 (3)	0.0660 (10)	
N8	−0.1258 (5)	0.23914 (15)	0.0977 (3)	0.0809 (13)	

### Atomic displacement parameters (Å^2^)

**Table:**

	*U*^11^	*U*^22^	*U*^33^	*U*^12^	*U*^13^	*U*^23^
C1	0.0501 (19)	0.0328 (18)	0.0338 (19)	−0.0032 (15)	0.0015 (15)	0.0035 (15)
C2	0.070 (3)	0.036 (2)	0.069 (3)	0.0021 (19)	0.007 (2)	0.002 (2)
C3	0.112 (4)	0.035 (2)	0.079 (3)	−0.002 (3)	0.006 (3)	0.005 (2)
C4	0.110 (4)	0.053 (3)	0.070 (3)	−0.038 (3)	0.006 (3)	0.010 (2)
C5	0.067 (3)	0.069 (3)	0.064 (3)	−0.026 (2)	0.009 (2)	0.003 (2)
C6	0.056 (2)	0.042 (2)	0.052 (2)	−0.0072 (17)	0.0069 (18)	0.0007 (18)
C7	0.0369 (17)	0.0353 (19)	0.045 (2)	0.0016 (14)	0.0104 (15)	−0.0001 (15)
C8	0.0334 (16)	0.0365 (19)	0.0387 (19)	−0.0038 (14)	0.0042 (14)	−0.0004 (15)
C9	0.0413 (18)	0.0328 (18)	0.044 (2)	−0.0093 (15)	0.0064 (15)	0.0002 (16)
C10	0.044 (2)	0.039 (2)	0.059 (2)	−0.0075 (16)	0.0116 (17)	−0.0016 (17)
C11	0.051 (2)	0.034 (2)	0.050 (2)	−0.0021 (17)	0.0028 (17)	0.0064 (17)
N1	0.0324 (13)	0.0315 (15)	0.0451 (17)	−0.0031 (12)	0.0045 (12)	0.0013 (13)
N2	0.0506 (17)	0.0364 (17)	0.085 (3)	−0.0009 (14)	0.0238 (17)	−0.0085 (16)
N3	0.0413 (18)	0.066 (2)	0.127 (4)	−0.0080 (17)	0.030 (2)	−0.011 (2)
N4	0.078 (2)	0.038 (2)	0.089 (3)	−0.0181 (18)	0.010 (2)	0.0034 (18)
C12	0.057 (2)	0.0302 (19)	0.042 (2)	−0.0028 (16)	−0.0046 (17)	0.0012 (16)
C13	0.060 (2)	0.048 (2)	0.057 (3)	−0.0086 (19)	0.010 (2)	0.0004 (19)
C14	0.085 (3)	0.058 (3)	0.077 (3)	−0.026 (2)	0.013 (3)	−0.002 (2)
C15	0.109 (4)	0.060 (3)	0.070 (3)	−0.038 (3)	−0.005 (3)	0.007 (3)
C16	0.125 (4)	0.022 (2)	0.084 (4)	−0.002 (3)	−0.018 (3)	0.002 (2)
C17	0.078 (3)	0.040 (2)	0.067 (3)	0.009 (2)	−0.006 (2)	−0.006 (2)
C18	0.0454 (19)	0.044 (2)	0.045 (2)	0.0033 (16)	0.0091 (16)	−0.0035 (17)
C19	0.0361 (16)	0.0337 (19)	0.043 (2)	−0.0032 (14)	0.0058 (15)	0.0029 (15)
C20	0.0466 (19)	0.0349 (19)	0.043 (2)	−0.0065 (15)	0.0083 (16)	−0.0014 (16)
C21	0.0450 (19)	0.038 (2)	0.051 (2)	−0.0029 (16)	0.0064 (17)	−0.0022 (17)
C22	0.054 (2)	0.041 (2)	0.070 (3)	−0.0045 (18)	0.017 (2)	−0.006 (2)
N5	0.0410 (15)	0.0309 (15)	0.0456 (17)	−0.0022 (12)	0.0050 (13)	−0.0021 (13)
N6	0.0520 (18)	0.0287 (16)	0.093 (3)	0.0009 (14)	0.0315 (18)	−0.0022 (16)
N7	0.0443 (18)	0.070 (2)	0.088 (3)	−0.0002 (17)	0.0241 (18)	0.002 (2)
N8	0.072 (2)	0.046 (2)	0.127 (4)	−0.0218 (19)	0.026 (2)	−0.005 (2)

### Geometric parameters (Å, °)

**Table:**

C1—C2	1.381 (5)	C12—C13	1.387 (5)
C1—C6	1.389 (5)	C12—C17	1.398 (5)
C1—C7	1.464 (4)	C12—C18	1.457 (5)
C2—C3	1.387 (6)	C13—C14	1.362 (5)
C2—H2	0.9300	C13—H13	0.9300
C3—C4	1.365 (7)	C14—C15	1.375 (6)
C3—H3	0.9300	C14—H14	0.9300
C4—C5	1.369 (6)	C15—C16	1.370 (7)
C4—H4	0.9300	C15—H15	0.9300
C5—C6	1.378 (5)	C16—C17	1.384 (6)
C5—H5	0.9300	C16—H16	0.9300
C6—H6	0.9300	C17—H17	0.9300
C7—N1	1.266 (4)	C18—N5	1.276 (4)
C7—H7	0.9300	C18—H18	0.9300
C8—C9	1.360 (4)	C19—C20	1.360 (4)
C8—N1	1.387 (4)	C19—N5	1.382 (4)
C8—C10	1.437 (4)	C19—C21	1.434 (4)
C9—N2	1.345 (4)	C20—N6	1.344 (4)
C9—C11	1.443 (4)	C20—C22	1.446 (5)
C10—N3	1.136 (4)	C21—N7	1.139 (4)
C11—N4	1.138 (4)	C22—N8	1.134 (4)
N2—H2A	0.8600	N6—H6A	0.8600
N2—H2B	0.8600	N6—H6B	0.8600
			
C2—C1—C6	119.5 (3)	C13—C12—C17	118.9 (4)
C2—C1—C7	119.2 (3)	C13—C12—C18	121.2 (3)
C6—C1—C7	121.3 (3)	C17—C12—C18	119.9 (4)
C1—C2—C3	119.9 (4)	C14—C13—C12	121.4 (4)
C1—C2—H2	120.1	C14—C13—H13	119.3
C3—C2—H2	120.1	C12—C13—H13	119.3
C4—C3—C2	120.1 (4)	C13—C14—C15	119.2 (5)
C4—C3—H3	119.9	C13—C14—H14	120.4
C2—C3—H3	119.9	C15—C14—H14	120.4
C3—C4—C5	120.3 (4)	C16—C15—C14	121.1 (4)
C3—C4—H4	119.8	C16—C15—H15	119.5
C5—C4—H4	119.8	C14—C15—H15	119.5
C4—C5—C6	120.3 (4)	C15—C16—C17	120.1 (4)
C4—C5—H5	119.8	C15—C16—H16	120.0
C6—C5—H5	119.8	C17—C16—H16	120.0
C5—C6—C1	119.8 (4)	C16—C17—C12	119.4 (4)
C5—C6—H6	120.1	C16—C17—H17	120.3
C1—C6—H6	120.1	C12—C17—H17	120.3
N1—C7—C1	121.8 (3)	N5—C18—C12	121.4 (3)
N1—C7—H7	119.1	N5—C18—H18	119.3
C1—C7—H7	119.1	C12—C18—H18	119.3
C9—C8—N1	118.1 (3)	C20—C19—N5	117.3 (3)
C9—C8—C10	119.6 (3)	C20—C19—C21	119.9 (3)
N1—C8—C10	122.3 (3)	N5—C19—C21	122.9 (3)
N2—C9—C8	124.0 (3)	N6—C20—C19	124.3 (3)
N2—C9—C11	115.7 (3)	N6—C20—C22	116.1 (3)
C8—C9—C11	120.3 (3)	C19—C20—C22	119.6 (3)
N3—C10—C8	176.8 (4)	N7—C21—C19	178.4 (4)
N4—C11—C9	178.2 (4)	N8—C22—C20	179.1 (4)
C7—N1—C8	121.0 (3)	C18—N5—C19	121.4 (3)
C9—N2—H2A	120.0	C20—N6—H6A	120.0
C9—N2—H2B	120.0	C20—N6—H6B	120.0
H2A—N2—H2B	120.0	H6A—N6—H6B	120.0

### Hydrogen-bond geometry (Å, °)

**Table:**

*D*—H···*A*	*D*—H	H···*A*	*D*···*A*	*D*—H···*A*
N2—H2A···N3^i^	0.86	2.36	3.096 (4)	144
N2—H2B···N8^ii^	0.86	2.25	3.090 (5)	165
N6—H6A···N7^iii^	0.86	2.51	3.228 (5)	142
N6—H6B···N4^iv^	0.86	2.28	3.057 (4)	150

Symmetry codes: (i) *x*−1, *y*, *z*; (ii) −*x*, *y*+1/2, −*z*+1/2; (iii) *x*+1, *y*, *z*; (iv) −*x*+1, *y*−1/2, −*z*+1/2.
